# Simulation studies for characterizing ultrashort bunches using novel polarizable X-band transverse deflection structures

**DOI:** 10.1038/s41598-019-56433-8

**Published:** 2019-12-27

**Authors:** Daniel Marx, Ralph W. Assmann, Paolo Craievich, Klaus Floettmann, Alexej Grudiev, Barbara Marchetti

**Affiliations:** 1 DESY, Notkestraße 85, 22607 Hamburg, Germany; 2 Department of Physics, Universität Hamburg, Hamburg, Germany; 3 Paul Scherrer Institut, Villigen, PSI Switzerland; 4 CERN, 1211, Genève, Switzerland

**Keywords:** Applied physics, Physics, Techniques and instrumentation

## Abstract

Transverse deflection structures are useful devices for characterizing the longitudinal properties of bunches in electron accelerators. With efforts to produce ever-shorter bunches for applications such as external injection into novel accelerator structures, e.g. plasma cells or dielectric structures, the applicability of deflection structures to measuring ultrashort bunches has been considered. In this paper, charge-density and bunch-length measurements of femtosecond and subfemtosecond bunches at the ARES linac at the SINBAD facility at DESY are studied with simulations and the limitations discussed in detail. The novel polarizable X-band transverse deflection structure (PolariX-TDS) will allow the streaking of bunches at all transverse angles, making a 3D charge-density reconstruction of bunches possible, in addition to the standard 1D charge-density reconstruction and bunch-length measurements. These various measurements of the charge-density distributions of bunches have been simulated, and it is shown that useful information about ultrashort bunches down to subfemtosecond lengths may be obtained using the setup planned for the ARES linac.

## Introduction

Transverse deflection structures (TDS) are widely-used devices in electron linacs for diagnosing bunches^1^. The principle is that the fields within a structure exert a time-dependent transverse force on a bunch. Such structures are typically operated at or near zero crossing of the phase, at which the head and tail of the bunch experience very different forces (in opposite directions in the case of zero crossing), and the bunch is therefore streaked transversely. This streaking is very useful for diagnostic purposes, as usually observation screens only provide information about the transverse profile. By streaking a bunch transversely and placing a screen downstream, information about the longitudinal profile of the bunch may be obtained.

Standard deflection structures allow beams to be streaked along a fixed transverse axis. The longitudinal distribution at the position of the TDS is mapped onto the streaking axis, allowing the distribution perpendicular to this axis to be obtained along the bunch. For example, by streaking a bunch in the vertical direction, the horizontal profile as a function of longitudinal position may be obtained. A novel travelling-wave deflector that allows the fields within to be rotated and therefore the angle of streaking to be varied, will be installed at several DESY facilities^2^. This structure, the polarizable X-band transverse deflection structure (PolariX-TDS), which is being designed and produced in a collaboration between CERN, DESY and PSI^3^, allows information about the transverse distribution in all planes to be obtained, which is particularly useful when dealing with transversely asymmetric bunches. These profiles may be combined using tomographic reconstruction techniques to generate a full 3D charge-density image of bunches^4^.

Measuring ultrashort bunches with a TDS is challenging and is limited by various factors that are explored in this paper. The measurement of sub-10 fs bunch lengths using an X-band TDS has already been demonstrated^5^; however, there are currently attempts to produce electron bunches with bunch lengths down to the subfemtosecond scale, including at the ARES linac^6^, which is under construction at the SINBAD facility at DESY^7^. In contrast to previous work published, the energy range considered around 100 MeV is also different, lying between the low-energy operation of a photoinjector^5,8^ and the higher-energy range of a few GeV typical of FEL facilities^9–12^, although in some cases it can be advantageous to measure the beam properties in FEL injectors at this energy. This energy is low enough that the beam is not rigid, so bunch properties evolve considerably along the beamline, due to both their energy distribution and space-charge forces. The beam is also very sensitive to the fields in the TDS, so small imperfections can result in a big orbit excursion. In order to simulate realistically the measurement process, both the field map of the TDS and 3D space-charge forces must be included.

The femtosecond-range bunch length and energy range of around 100 MeV at the ARES linac are appropriate for external injection into novel accelerating structures, such as plasma cells for laser wakefield plasma acceleration (LWFA)^13–15^ or dielectric gratings for dielectric laser acceleration (DLA)^16^. Being able to determine the properties of injected bunches is essential in order to investigate these new acceleration methods. Reliable bunch-length and longitudinal-profile measurements are necessary, as the fields within such novel accelerating structures are highly dependent on longitudinal position; hence the need for ultrashort bunches. In addition, after bunch compression using a magnetic chicane, the bunches can be very asymmetric due to the effects of coherent synchrotron radiation. Measuring the full 3D charge density is therefore very useful, as the shape of the bunch and correlations between planes can affect the focusing and accelerating fields. These measurements could potentially be used to optimize the parameters of the accelerator. Finally, measuring bunches after acceleration in these structures is very important for testing and optimizing these novel acceleration techniques.

This paper illustrates and explores the limitations of bunch-length and charge-density measurements with the PolariX-TDS, using ARES bunch properties as examples. Although the PolariX-TDS will be installed at several other facilities, including FLASHForward^17^ and FLASH2^18^ at DESY and the Athos beamline^19^ at SwissFEL, the ARES linac will operate with bunches at much lower energies and with much shorter bunch lengths than the other facilities, introducing unique challenges for diagnostic purposes, which are discussed here. The structure itself is introduced in the following section, and simulations of a single electron’s trajectory through the TDS are shown to illustrate deviations from the ideal case. The theory of beam dynamics in a TDS is presented, along with a brief introduction to ARES and the ultrashort bunches that will be diagnosed with the TDS. Thereafter come sections dedicated to simulations of charge-density measurements, which include both the simulated field maps and 3D space charge. The capability of reaching resolutions as low as 0.2 fs, which is lower than has so far been achieved experimentally, is demonstrated for beam characterization in a regime of particular importance for LWFA experiments. Simulations for 3D charge-density reconstructions, which in contrast to previous work include the effects mentioned above, are presented. Finally, considerations relating to calibration, manufacturing imperfections and jitter are discussed.

## A Novel Deflection Structure

**Figure 1:**
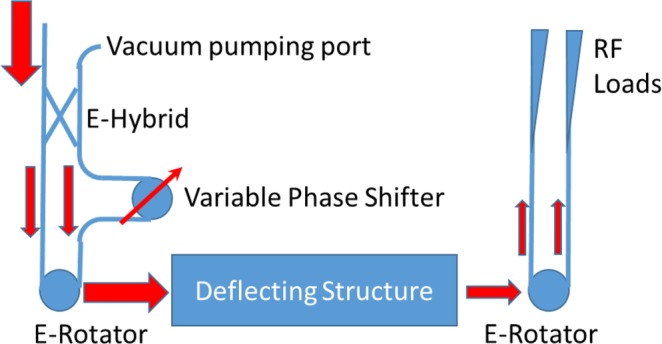
Schematic of the RF design for the TDS, including the E-rotator^20^.

A compact circular waveguide TE11 rotating mode launcher (E-rotator) was designed at CERN in 2016^20^. This component has several potential applications, including a linearly-polarized mode launcher, as illustrated in Fig. 1. An E-hybrid, which consists of four ports, splits the incoming power equally into two branches in the plane tangential to the electric field. Using a phase shifter situated on one branch, the phase difference between the modes in the two branches can be adjusted. The input waves are then coupled into an E-rotator, a three-port hybrid that combines two circularly-polarized modes rotating in opposite directions to form a linearly-polarized output mode if the same amount of power is injected into each input port. The orientation of this output mode is dependent on the phase difference of the two input modes and so can be controlled with the phase shifter.

### Structures

Two types of structures will be produced: a short structure of 96 regular cells and a long structure of 120 regular cells. The latter is currently only planned for installation at SwissFEL; the DESY facilities will use short structures. The power will be coupled into the structures at the downstream end; the power of the travelling wave will propagate backwards with the group velocity while the phase velocity propagates forwards at the speed of light. A matching cell is located at each end of the structure, between the coupler and the regular cells. The structures will be operated at slightly different frequencies at the various facilities and the water temperature will be used to adjust for the difference. Table 1 shows the key parameters for the short structure.

**Table 1:** Parameters for the short PolariX-TDS^2^.

Parameter	Value
Phase advance per cell (deg)	120
Iris radius (mm)	4
Iris thickness (mm)	2.6
Group velocity (%c)	$-$2.666
Quality factor	6490
Shunt impedance (M$\Omega {{\rm{m}}}^{-1}$)	50
Number of cells	96
Filling time (ns)	104.5
Active length (mm)	800
Total length (mm)	960
Power-to-voltage (MV/M${{\rm{W}}}^{0.5}$)	5.225

This new structure design requires high manufacturing precision to minimize deviations from azimuthal symmetry in the copper structure that would lead to the rotation of the polarized modes and therefore a deterioration of the field. Whereas the average kick can be rotated to the correct plane using the variable phase shifter, there will be a loss of the total effective kick due to this rotation. For this reason, the high-precision tuning-free production process developed for the C-band linac of the SwissFEL project^21^, which was also used for the fabrication of tuning-free X-band structure prototypes for CLIC^22^, is being used for the manufacture of the PolariX-TDSs.

### Fields and trajectory in structure

The electromagnetic fields inside a TDS can be described by a complex 3D field map comprising both electric and magnetic field components. The transverse deflecting force that an electron experiences while passing through the structure is given by

1 $$\overrightarrow{F}={\rm{Re}}\left[e(\overrightarrow{E}+\overrightarrow{v}\times \overrightarrow{B})\exp \left(i\left(\omega t+\phi \right)\right)\right],$$

where $\overrightarrow{E}$ and $\overrightarrow{B}$ are complex vectors of the electric and magnetic fields respectively, $\omega =2\pi f$ where $f$ is the RF frequency, $t$ is the time coordinate, and $\phi$ is a constant that defines the operational phase. Field maps in this format were generated using ANSYS HFSS software^23^.

The phase of the field on a particle is given by a combination of the phase of the field map and the time-dependent $i\omega t$ term. When the TDS is well tuned, the phase velocity within the regular cells of the structure is equal to the speed of light, and these two contributions approximately cancel, leading to an approximately constant phase. The tuning is achieved in practice by regulating the temperature of the TDS by means of the water temperature. The nominal operating temperature for the ARES frequency of 11.9916 GHz is 48 °C; however, the field maps were generated for the SwissFEL frequency of 11.9952 GHz, for which the nominal operating temperature is 30 °C. It was found that the differences resulting from using the fields calculated for the SwissFEL frequency and temperature rather than those at ARES are negligible.

The standard operating mode for a TDS used for diagnostic purposes is at zero crossing of the phase, i.e. when the bunch centre would, in the ideal case, experience no deflecting field and pass in a straight line through the structure. In a real TDS, it is not possible in general to find a phase at which an electron travels undeflected on a straight line; therefore, an electron exiting with zero angle with respect to the longitudinal axis would exit with a transverse offset from the axis^8^. In particular, in the PolariX-TDS, electrons are subject to unwanted kicks in the end matching cells, which are especially significant at the relatively low beam energies of ARES. These kicks are a side effect of a design that minimizes the surface fields in the coupler, which is important for high-power operation, such as at SwissFEL.

**Figure 2:**
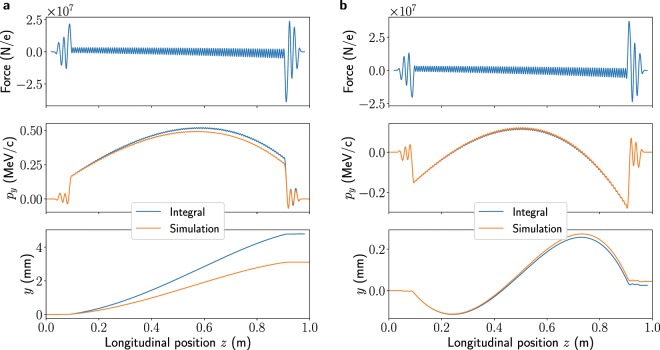
Vertical force, momentum and displacement for a 100 MeV electron at zero-phase crossing in the TDS for the field map corresponding to the nominal operating temperature (**a**) and 1.84 °C under the nominal operating temperature (**b**). The amplitude corresponds to an on-crest voltage kick of 20 MV along the $y$ axis. Both analytical calculations from the field map and ASTRA simulation results are shown. The scales are different for the two geometries.

Figure 2 shows the on-axis force calculated from the field map as a function of longitudinal position, along with the particle momentum and trajectory in the deflecting direction (taken to be $y$). The amplitude has been scaled such that the maximum kick received by an on-crest particle would be 20 MV, which is a conservative estimate of the maximum that can be expected at ARES after taking into account losses in the waveguides, and the particle energy is 100 MeV. The results from single-particle tracking through the field maps in ASTRA^24^ are also shown. For both methods, the zero phase, defined as the phase for which $y^{\prime} =0$ at the exit of the structure, has been found by scanning the phase. The results from the two methods agree very well and show that, at this deflecting voltage, the electron exits with a deviation of approximately 3 mm. Given that the aperture radius in the TDS is limited to 4 mm, this level of offset is not acceptable.

One way to compensate for this offset is by changing the temperature so that it is not exactly tuned to the regular cells. This has the effect of changing the geometry of the structure and therefore the phase velocity of the wave as it propagates through a cell. The largest effect can be seen in the end cells, although there will also be phase slippage in the regular cells. The resultant change in force with respect to the tuned case leads to a particle trajectory within the structure that can significantly reduce the offset caused by the entrance and exit kicks. In this case it was found that a −1.84 °C change in temperature almost entirely cancels the offset in trajectory, and field maps were generated for this new geometry. The equivalent results using these new field maps are also shown in Fig. 2. The final offset at 20 MV for a 100 MeV beam is now approximately 0.04 mm. It was therefore decided to operate the structures with this temperature offset, and the simulations of measurements in the rest of this paper have been performed using these field maps.

When operating these structures in the beamline, it will be necessary to determine the ideal operating temperature experimentally. A beam position monitor will be installed downstream of the TDS, which may be used in combination with the screen to find the zero-crossing phase. The temperature must be adjusted to find the set point that minimizes the beam deviation at zero crossing, which involves resetting the phase for each new temperature set point. The target temperature stability at ARES is 0.02 °C.

As well as the transverse fields in the field map, there are also longitudinal fields, which vary according to transverse position. This is a necessary consequence of the Panofsky-Wenzel theorem^25^. At sub-ultrarelativistic velocities when the effects of differing velocities cannot be neglected, such as at the ARES linac, these longitudinal fields will therefore perturb the particle distributions.

## Beam-Dynamics Theory

A TDS imparts a time-dependent transverse kick on an electron bunch. In the following analysis, it is assumed that this kick is in the $y$ direction and the TDS is operated at zero crossing. The distribution in $y$ is modified and the longitudinal distribution of the bunch at the position of the TDS is imprinted on the $y$ profile. The resultant distribution on a screen located downstream at longitudinal position ${z}_{1}$ is assumed to be a convolution of the original distribution in $y$ when the TDS is switched off and the longitudinal distribution mapped onto the $y$ axis. If these two contributions are uncorrelated, their variances add in quadrature to give the combined spot size at the screen^26^:

2 $${\sigma }_{y}({z}_{1})=\sqrt{{({\sigma }_{y}^{{\rm{off}}}({z}_{1}))}^{2}+{({S}_{y}c{\sigma }_{t})}^{2}}.$$

Here, ${\sigma }_{y}^{{\rm{off}}}$ is the rms spot size in $y$ with the TDS off, ${\sigma }_{t}$ is the rms bunch length at the centre of the TDS in seconds, $c$ is the speed of light and ${S}_{y}$ is the shear parameter, which relates the shift in position of a particle on the screen, $\Delta y$, to its position along the bunch relative to the central particle, $\zeta$:

3 $$\Delta y\approx {S}_{y}\zeta .$$

The divergence in the streaking direction when the TDS is operated at zero crossing is^10^

4 $$\Delta y^{\prime} (\zeta )=\frac{e{V}_{0}}{c| p| }\ \sin \left(2\pi f\zeta /c\right)\approx \frac{2\pi fe{V}_{0}}{{c}^{2}| p| }\zeta ,$$

where $f$ and ${V}_{0}$ are the TDS frequency and peak voltage respectively, and $p$ is the mean momentum. A matrix transformation may be applied to convert the vertical particle coordinates from the centre of the TDS, ${z}_{0}$, to the screen, ${z}_{1}$, when the TDS is off:

5 $$\left(\begin{array}{c}y\ \left({z}_{1}\right)\\ y^{\prime} \left({z}_{1}\right)\end{array}\right)=\left(\begin{array}{cc}{M}_{1,1}^{y} & {M}_{1,2}^{y}\\ {M}_{2,1}^{y} & {M}_{2,2}^{y}\end{array}\right)\left(\begin{array}{c}y\ \left({z}_{0}\right)\\ y^{\prime} \left({z}_{0}\right)\end{array}\right).$$

With the TDS on, the particle position on the screen is shifted by

6 $$\Delta y(\zeta )={M}_{1,2}^{y}\Delta {y}^{^{\prime} }(\zeta )\approx {S}_{y}\zeta ,$$

where the shear parameter is defined as

7 $${S}_{y}={M}_{1,2}^{y}\frac{2\pi fe{V}_{0}}{{c}^{2}| p| },$$

where ${M}^{y}$ is the vertical transfer matrix from the centre of the TDS to the screen^10^.

In general,

8 $${M}_{1,2}^{y}=\sqrt{{\beta }_{y}({z}_{0}){\beta }_{y}({z}_{1})}\sin \Delta {\phi }_{y},$$

where ${\beta }_{y}({z}_{0})$ and ${\beta }_{y}({z}_{1})$ are the $\beta$-functions at the centre of the TDS and the screen respectively, and $\Delta {\phi }_{y}$ is the phase advance between these two locations. The temporal resolution is defined here as the bunch length in seconds when the two terms on the right-hand side of Eq. (2) are equal, and can therefore be expressed as

9 $${R}_{{\rm{t}}}=\frac{{\sigma }_{y}^{{\rm{off}}}({z}_{1})}{{S}_{y}c}\approx \frac{\sqrt{{\epsilon }_{y}}}{\sqrt{{\beta }_{y}({z}_{0})}| \sin (\Delta {\phi}_{y})| }\frac{| p| c}{2\pi fe{V}_{0}},$$

where ${\epsilon }_{y}$ is the vertical geometric emittance of the bunch.

It is useful to consider the meaning of the resolution defined here. If the time slices are much larger than the resolution, then the contribution to the spot size at the screen from the streaking is much more significant than the contribution from the transverse particle distribution before the TDS. It is therefore only possible to resolve differences in features between time slices that are larger than the resolution. This does not mean, however, that a measurement of the rms bunch length, where this is smaller than the resolution, is not possible. The bunch length measurement does not, in theory, rely on the ability to reconstruct accurately the features of the beam, but instead relies on the addition of variances as shown in Eq. (2). It therefore requires a measurement of the spot sizes with the TDS on and off, which in experiment could be averaged over several shots to reduce the effects of jitter on the measurement. Nevertheless, this measurement is limited by the uncertainties in the measurement of the spot sizes, including the imaging system. A good longitudinal resolution (as defined above) can therefore minimize these sources of uncertainty in the bunch-length measurement. In addition to the betatron beam size, which is encompassed in the resolution defined here, there are many other sources of discrepancies in a reconstruction that are considered in this paper.

## The ARES Linac

**Figure 3:**
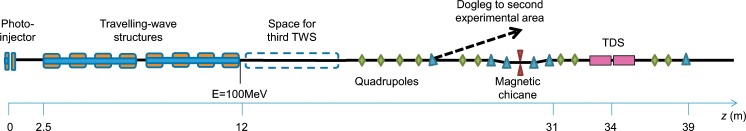
Schematic of the ARES linac before the installation of plasma experiments.

The ARES linac at SINBAD is a linear electron accelerator, which will produce suitable beams for experiments investigating plasma-wakefield and dielectric acceleration. The accelerator is being constructed in phases and it is expected that two PolariX-TDSs will be installed at the end of the main beamline in 2020. A schematic of the future linac is shown in Fig. 3. A photoinjector accelerates the beam to around 5 MeV and is followed by two travelling-wave structures (TWS), which bring the beam energy up to around 100 MeV while manipulating the longitudinal phase space as required for bunch compression (a third TWS will be installed in a future upgrade). A series of six quadrupoles, which will be used for matching the beam, is followed by a magnetic chicane for compressing bunches (compression by pure velocity bunching in the TWSs is also possible). The space downstream of the chicane will be dedicated initially to beam diagnostics with two deflection structures in sequence, surrounded by two quadrupoles on each side, along with screens and other diagnostic devices. A longer length of deflecting structure results in a greater streaking force and having two structures rather than one reduces the power dissipation and phase slippage along each structure. This section of the beamline will be useful during commissioning of the accelerator to measure the longitudinal bunch profile as well as to see, for instance, the effects of wakefields or nonlinearities on the bunches. This will provide valuable information about the properties of bunches that will eventually be injected into the plasma. It is planned to move the TDS and other diagnostics further downstream after the plasma experimental area when this is installed at a later date. Using this beamline, it will be possible to measure the properties of the bunches produced after acceleration in the plasma cells as well as the properties with the plasma off. Due to the bunch evolution, it will be challenging to reconstruct exactly the distribution at the plasma injection point; nevertheless, the diagnostics beamline will provide useful information about the properties of the bunches.

The ARES linac may be tuned in various ways and so provides the flexibility to produce bunches with a sizeable range of properties^27^. The bunches used for the simulations presented here have been generated in start-to-end simulations of the ARES linac.

### Subfemtosecond bunches

Using pure magnetic compression, it should be possible to achieve subfemtosecond bunch lengths at ARES while aiming for an arrival-time jitter of around 10 fs. WP1^27^ is an example of such a working point. The bunch length at the chicane exit is 0.51 fs, which makes it a good working point for studying the measurement of ultrashort bunches and resolution constraints. In addition, although the charge is relatively low (0.79 pC), the short bunch length means that the peak current of 2.1 kA at the chicane exit is high compared to other working points, so studying this working point should give an idea of how much space charge interferes with the measurement.

**Figure 4:**
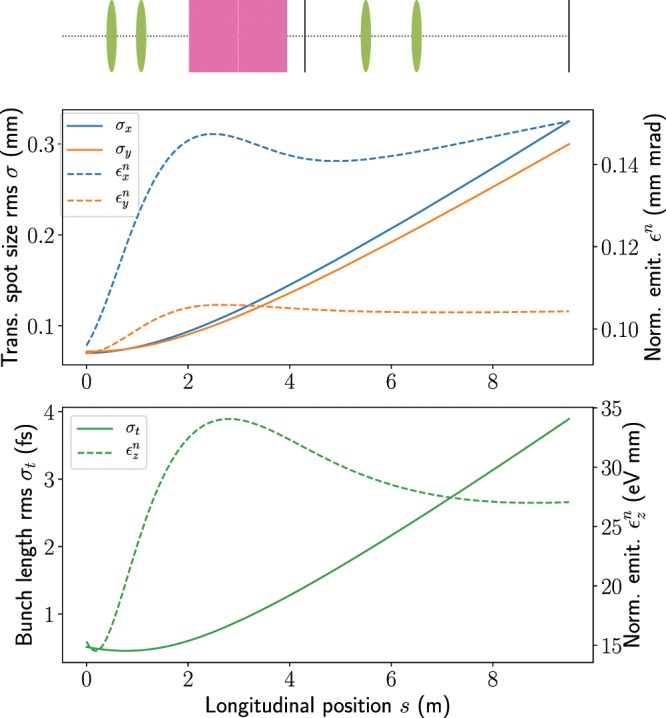
Evolution of bunch sizes and emittance from the exit of the chicane to the final screen after the TDS assuming a drift length for the subfemtosecond bunch. The positions of the TDSs, quadrupoles and screens are indicated for reference, although these elements have not been included in the simulation. The simulation was performed in ASTRA with 3D space-charge forces included.

**Figure 5:**
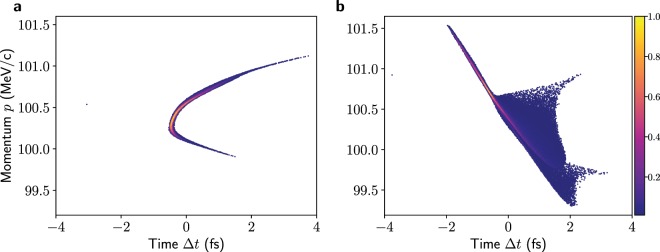
Longitudinal phase-space distributions at the exit of the chicane (**a**) and between the two structures (**b**) with the TDSs off. The spread in the latter distribution arises due to space-charge forces.

The bunch distribution for this working point at the exit of the chicane was transported onwards with 3D space-charge effects included in ASTRA. The evolution of the bunch dimensions and emittance are illustrated in Fig. 4 from the exit of the chicane to the final screen, with both TDSs and all quadrupoles turned off. The bunch properties at the exit of the chicane and in between the two TDSs are summarized in Table 2. Although initially the bunch length decreases due to the effects of velocity bunching, the phase space evolves in such a way that the length quickly starts to increase and the increase within the two TDSs is significant. The longitudinal phase space distributions at the exit of the chicane and at the centre of the two TDSs (which are turned off) are shown in Fig. 5. In other work using deflection structures below ultrarelativistic energies, the accelerator settings have been been adjusted to achieve a focus at the centre of the TDS^5^. This approach is not adopted for the studies in this paper, as the aim is to be able to diagnose bunches while avoiding modifying them as much as possible.

**Table 2:** Bunch properties at the exit of the chicane and in between the two structures with the TDS and quadrupoles off for the subfemtosecond bunch.

Parameter	Chicane exit	Between structures
Mean energy (MeV)	100	100
Bunch charge (pC)	0.79	0.79
Bunch length rms (fs)	0.510	0.890
Rel. rms energy spread	0.0019	0.0042
Hor. rms beam size (mm)	0.069	0.117
Ver. rms beam size (mm)	0.072	0.109
Norm. hor. emittance (mm mrad)	0.096	0.146
Norm. ver. emittance (mm mrad)	0.094	0.106
Horizontal $\beta$-function (m)	9.7	18.3
Vertical $\beta$-function (m)	10.8	21.9

Downstream of the TDSs, the evolution in the longitudinal distribution is not so important for the purposes of the measurement, as the longitudinal axis has already been mapped to a transverse axis, and so it only plays a role insofar as there is coupling with the transverse axes, for example due to space charge effects. A significant increase in the emittance, especially in the horizontal plane, is also visible between the exit of the chicane and the TDS. The temporal resolution is a function of the emittance in the streaking plane so the difference in emittance between the two planes could be a good reason to streak the beam in the $y$ direction.

## 1D Charge-Density Reconstruction and Bunch-Length Measurement

Measuring the bunch length and reconstructing the 1D longitudinal charge-density profile are standard measurements using a TDS. Using the shear parameter, which can be obtained from a phase scan, the $y$ coordinate on the screen can be converted to the position along the bunch, $\Delta t$, in case of streaking along the $y$-axis. The second screen downstream of the TDS is used for the reconstructions presented here in order to optimize the phase advance. By summing over all pixels for a constant $y$, a histogram of the longitudinal charge profile along the bunch can be reconstructed. If the bunch length has a similar magnitude to the longitudinal resolution, it is necessary to subtract the betatron beam size when calculating the bunch length. From Eq. (2), one obtains

10 $${\sigma }_{t}=\frac{\sqrt{{\sigma }_{y}^{2}({s}_{1})-{\left({\sigma }_{y}^{{\rm{off}}}\right)}^{2}}}{Sc}.$$

In this section, we show with realistic simulations how this method can be successfully applied to subfemtosecond bunches and discuss the limitations.

### Optimization of quadrupole strengths

There are various quadrupoles that may be used to optimize the beam properties for this measurement, including two directly upstream of the TDSs and two downstream. To avoid interfering with the distribution before the TDS, it was decided to use the quadrupoles after the TDSs for matching for this measurement. Analytically, a $\pi /2$ phase advance between the centre of the TDSs and the screen is desired, according to Eq. (9), and this could be set as an optimization constraint for a matrix transformation, for instance in ELEGANT^28^. Assuming this phase advance, a resolution of 0.16 fs is possible according to Eq. (9), where the emittance and $\beta$-function have been determined from simulations of a drift length in ASTRA with space-charge effects included.

In an experimental scenario, the phase advance is not directly measurable, as it depends on the $\beta$-function throughout the beamline. In addition, space-charge effects are significant for the bunches considered. Therefore, tracking simulations in ASTRA incorporating 3D space-charge forces are necessary. For the optimization, it is easier to consider minimizing the ratio of the spot size on the screen with the TDS off to that with the TDS on: ${\sigma }_{y}^{{\rm{off}}}({s}_{1})/{\sigma }_{y}({s}_{1})$. This can be related to the longitudinal resolution with the equation

11 $$\frac{{\sigma }_{y}^{{\rm{off}}}({z}_{1})}{{\sigma }_{y}({z}_{1})}=\frac{1}{\sqrt{1+{\left(\frac{{\sigma }_{t}}{{R}_{t}}\right)}^{2}}}.$$

This equation shows that optimizing the longitudinal resolution results in the minimum possible ratio. When using only quadrupoles downstream of the deflectors, it is clear from Eq. (9) that minimizing this ratio by scanning the quadrupole strengths results in a phase advance approaching $\pi /2$. For the bunch length and minimum resolution quoted above, this equation gives a value of 0.17 for the minimum ratio.

**Figure 6:**
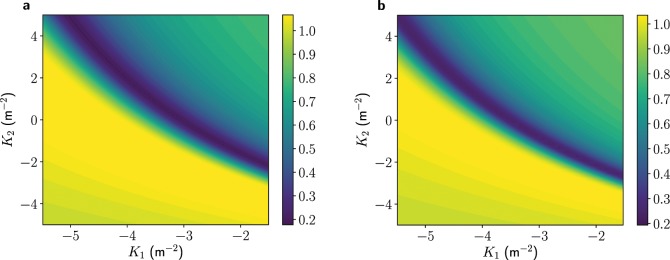
Ratio ${\sigma }_{y}^{{\rm{off}}}({s}_{1})/{\sigma }_{y}({s}_{1})$ shown as a colour map for various quadrupole focusing strengths of the two quadrupoles, ${K}_{1}$ and ${K}_{2}$. Results from ASTRA simulations with space-charge effects excluded (**a**) and included (**b**). The fields were scaled such that each TDS would provide an on-peak voltage deflection of 20 MV, although they are operated at zero-crossing here.

A scan over the two quadrupole strengths, ${K}_{1}$ and ${K}_{2}$, was performed in ASTRA to find their optimal settings. This is analogous to the procedure that will be used in the control room when operating. Figure 6 shows the results of this scan, with and without space-charge forces from the first TDS onwards. It can be seen that there are various combinations of quadrupole settings that can minimize this ratio. The only other constraint is ensuring that the beam size fits on the screen and is large enough that it is not significantly limited by the screen resolution. Space-charge forces act in the transverse plane primarily as a defocusing lens, which explains why the ratio changes when these forces are included in simulations. There are, however, higher-order nonlinear components of these forces, which cannot be offset by the quadrupoles, and so the minimum ratio obtained when including space-charge effects is typically worse.

With ${K}_{1}$ fixed to $-3.04\ {{\rm{m}}}^{-2}$, a scan of ${K}_{2}$ was performed in ASTRA. A minimum ratio of 0.17 was achieved with a value of ${K}_{2}=-\,0.02\ {{\rm{m}}}^{-2}$ for simulations excluding space-charge forces, while a minimum ratio of 0.19 was achieved with a value of ${K}_{2}=-\,0.76\ {{\rm{m}}}^{-2}$ when including space-charge forces. For this ultrashort bunch, these ratios correspond to longitudinal resolutions of 0.16 fs and 0.18 fs respectively. This shows that the theoretical longitudinal resolution is not greatly worsened by the inclusion of space-charge effects.

### Simulations of measurements

**Figure 7:**
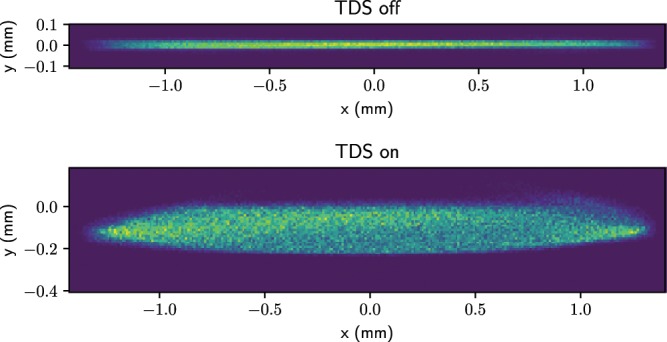
Screen profile images from ASTRA simulations with the TDS off (above) and on (below).

In this section, ASTRA simulations of the measurement are presented, including space-charge effects. The effects of wakefields were not included in these simulations, as analytical considerations indicated that these effects should not be significant at the low charges considered. Simulations were performed with 158,000 particles. Figure 7 shows the screen profiles for the TDS off and on. The resolution of the screen and imaging system combined has been taken to be $10\ {\rm{\mu }}{\rm{m}}$ for these simulations (based on scintillating screen stations designed for the European XFEL^29^). The shear parameter obtained from a phase scan is 219, corresponding to a longitudinal resolution of 0.18 fs, for fields scaled to effect a 20 MV on-crest kick per structure.

As discussed above, a bunch leaving one of the TDSs with $y^{\prime} =0$ will have an offset in the streaking direction. When this bunch passes through a quadrupole, it will be misaligned with respect to the axis of the quadrupole and therefore it will experience a transverse kick. This means that a bunch that exits the second TDS with $y^{\prime} =0$ will have a non-zero ${y}^{^{\prime} }$ at the screen. This should not cause an issue, assuming the bunch still arrives on the screen, as long as the relationship between arrival time (or phase) in the structure and transverse position on the screen in the direction of streaking remains linear. In this case, it can be seen in Fig. 7 that the displacement at the screen is small and should not be problematic.

**Figure 8:**
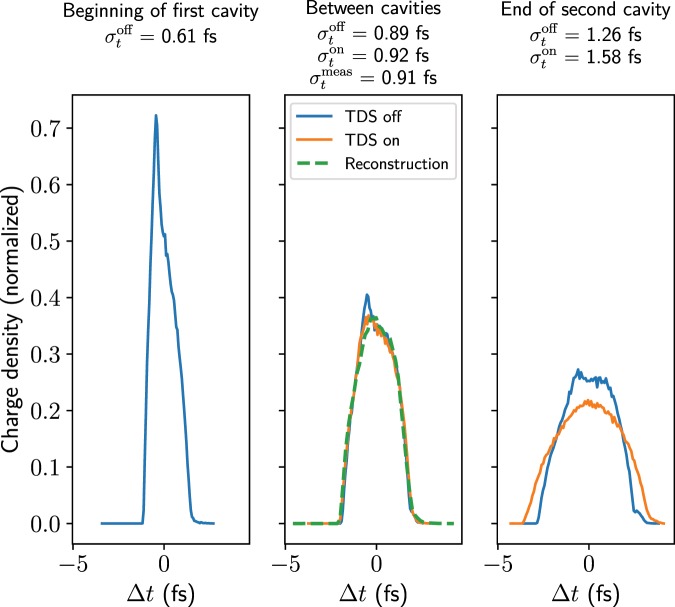
Reconstructed longitudinal charge profile shown alongside the actual longitudinal profiles from simulation, which are shown for both the TDS off and on. The rms bunch lengths of the simulated profiles and reconstruction, including betatron subtraction, are displayed above. The longitudinal resolution, which only includes the discrepancy due to the betatron beam size, is approximately 0.18 fs.

Figure 8 shows the reconstructed longitudinal charge distribution (obtained using the procedure detailed above) along with the actual longitudinal profiles from simulations at various points with the TDSs off and on. These show that the longitudinal profile is changing significantly in the space occupied by the two TDSs and also that the fields inside further distort the longitudinal profile, such that by the end of the second structure the profiles with and without TDS fields are fairly different. Nevertheless, the reconstructed bunch profile agrees very well with the simulated bunch profile between the two deflectors. The bunch length calculated is 0.926 fs directly from the screen image and 0.907 fs when subtracting the betatron function. One pixel corresponds to approximately 0.15 fs, so the measurement uncertainty is at the minimum half of this value. Given this, the value agrees well with the bunch length at the centre of the TDSs and, in fact, this number lies between the bunch lengths at the centre of the structures with the TDS off and with the TDS on.

Given that the ARES linac has two deflection structures that are independently powered, it is also possible to operate with just one at a time, which allows the evolution of the profiles to be measured. The longitudinal beam profile is in theory reconstructed at the position of the centre of the streaking, so operating the TDSs one at a time provides information about the evolution of the beam. As the kick imparted on a bunch when operating with one structure is only half as strong as when operating with two, the resolution is correspondingly decreased, resulting in a less-precise reconstruction.

Although this measurement could, in theory, be performed on a single electron bunch in a single shot (after phase scans have been performed) by streaking a bunch in perpendicular directions in the two deflectors, it is likely that the perturbation of the bunch profile due to the fields in the first TDS would prevent a fair comparison of the profiles streaked in the two directions. Optimizing the quadrupole strengths to achieve the correct phase advance (or minimum ratio of spot sizes) in both directions would be another challenge, especially in experiment. For these reasons, this approach has not been assumed in the simulations presented here but rather two separate (but identical) bunches are streaked along the same axis in different shots.

**Figure 9:**
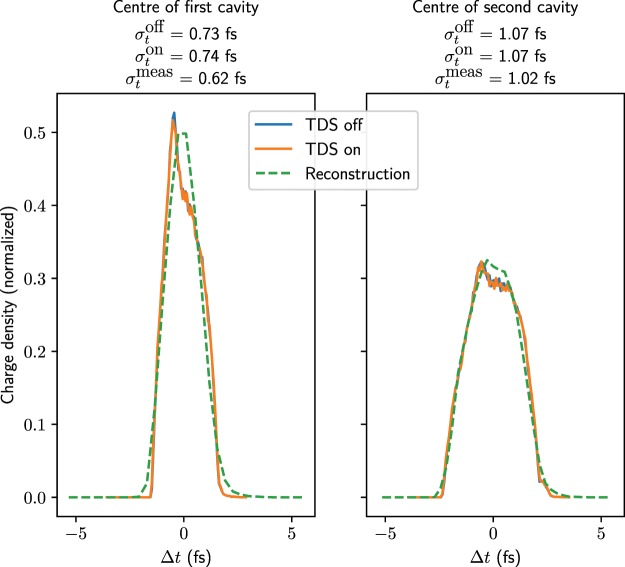
Reconstructed longitudinal charge profiles obtained with one TDS on at a time, shown superposed on the actual longitudinal profiles from simulation, which are shown for both the TDS off and on. The rms bunch lengths of the simulated profiles and reconstruction are displayed above. The longitudinal resolutions are 0.44 fs for the first TDS and 0.32 fs for the second TDS.

Although the quadrupole strengths would in theory need to be adjusted to optimize the resolution as the positions of the centres of the kicks are different from the case with two structures, for reasons of simplicity of the measurement it was decided to keep the quadrupole strengths the same. Repeating the phase scan with just one TDS on at a time is required nevertheless as the shear parameters will be different. Here, the shear parameters were determined to be 92.1 and 123.5 for the first and second TDS on respectively, corresponding to longitudinal resolutions of 0.44 fs and 0.32 fs. The bunch length measured in simulations with the first deflector on was (0.62 $\pm$ 0.18) fs and that with the second deflector on was (1.02 $\pm$ 0.14) fs, where the uncertainty values are taken as half of the pixel size converted to time using the shear parameter. The reconstructed profiles are shown in Fig. 9. Although the resolution is much worse than when using two structures and correspondingly the reconstruction is less precise, the increase in bunch length can be seen clearly, even with this extremely short bunch. For certain working points at ARES this will be a very useful measurement, especially given the fact that the beam evolves significantly in a fairly short distance.

### Limitations of measurement

The simulations of the measurements above provide very good agreement given the challenging regime of operation. In this section, the limitations of the measurement are explored and the various contributions to the discrepancy between the simulated longitudinal profile and the reconstruction are discussed. Table 3 gives explanations for the main sources of discrepancy in the measurement.

**Table 3:** Sources leading to discrepancy in the longitudinal profile measurement.

Source	Explanation
Finite transverse beam size	The transverse beam size limits the longitudinal resolution of the longitudinal profile reconstruction. Correlation between the transverse streaking direction and the longitudinal direction interferes with the measurement, although this can be accounted for by streaking at both zero crossings^9^.
Evolution of longitudinal profile within TDS	The bunch evolves as it is being streaked transversely, smearing the resulting distribution.
Longitudinal fields in TDS	The bunch is streaked longitudinally as different transverse slices of the beam are subject to different longitudinal forces, perturbing the longitudinal bunch profile within the TDS and leading to evolution of the longitudinal profile^8^. There is coupling between the two planes, making the assumption that they are uncorrelated invalid. These fields have a large effect on the reconstruction as can be seen in Fig. 11.
Chromaticity	The correlation between the energy and the transverse streaking direction induced by the longitudinal fields leads to chromatic effects when focusing in quadrupoles, distorting the transverse beam profile.
Space charge	The longitudinal bunch profile evolves due to longitudinal space charge forces within the TDS. Transverse space charge forces affect the transverse profile within and after the TDS. The phase scan is, however, not affected, leading to errors when reconstructing the original distribution. The effects of these forces may be seen in Fig. 11.
Imperfect TDS	Nonlinear transverse fields may contribute to a position-dependent streaking force in TDS. Boundary fields outside of normal cells lead to unwanted effects. Manufacturing imperfections may worsen results.
Screen resolution	Screen and imaging system limited by resolution (around $10\ {\rm{\mu }}{\rm{m}}$).

In order to evaluate the contribution of these effects to the measurement, several simulations were carried out using ELEGANT. For these simulations, rather than using field maps of the structure, a generic RF travelling-wave deflection structure was used (the RFDF element). This provides a deflection force that is constant as a function of transverse coordinates. The number of kicks given to the beam within the structure may be specified, which allows the ideal case considered in the theory Section above to be simulated, in which a single, instantaneous transverse kick is applied at the centre of the TDS or, in this case, between the two TDSs. Space-charge forces are not included in these simulations.

**Figure 10:**
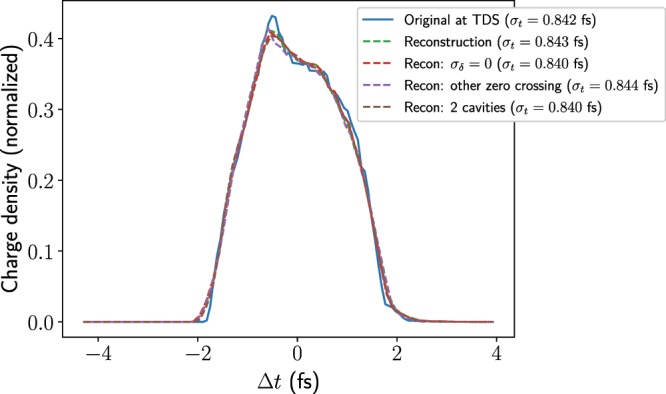
Comparison of the reconstructed longitudinal charge profiles from ELEGANT simulations for various cases. The longitudinal resolution is 0.17 fs.

Figure 10 shows the results of these ELEGANT simulations, taking the same input distribution before the first TDS as in the ASTRA simulations above. The actual longitudinal distribution between the two deflectors is shown in blue with no transverse kick applied. The reconstruction of the bunch receiving a single kick is also shown (green line). In order to see the effects of chromaticity, the energy spread of the beam distribution has been set to zero (${\sigma }_{\delta }=0$) and the reconstruction repeated (red line). The effect of initial correlation in the $y$-$t$ plane is investigated by streaking at the other zero crossing (purple line). Finally, two long deflectors are simulated with multiple kicks to see the effect of phase slippage and the evolution of the bunch, which is influenced by the induced energy spread caused by the longitudinal fields within the structures (brown line). The rms bunch lengths of these profiles are displayed in the legend. The spot size at the screen with the TDS off has been subtracted to obtain these values for the simulated measurement. All of these reconstructions show a similar agreement with the actual profile at the TDS, indicating that these various effects considered are small compared to that of the transverse beam size. It should be noted that, although the effect $y$-$t$ correlation is small in this case, it is possible to correct for it in cases when it cannot be neglected by streaking at both zero crossings^9^. For these simulations, the spot size at the screen with the TDS off is $13.2\ {\rm{\mu }}{\rm{m}}$, giving a longitudinal resolution of 0.17 fs.

**Figure 11:**
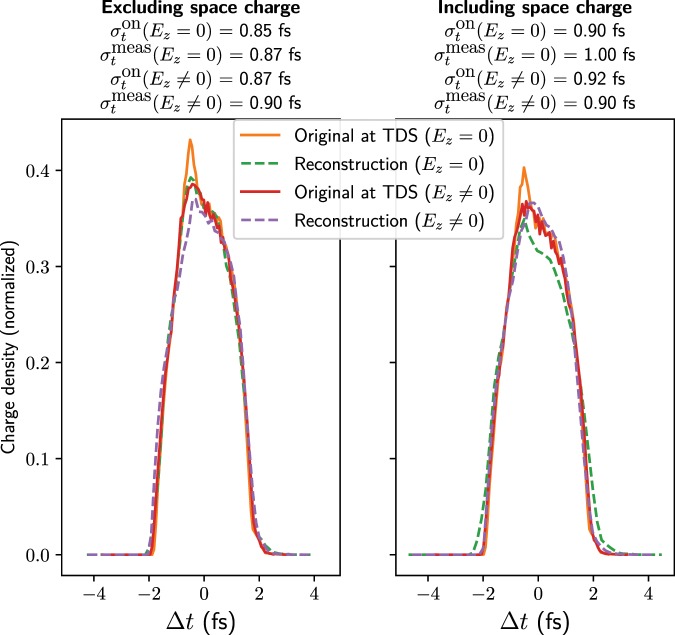
Reconstructed longitudinal charge profiles from ASTRA simulations. Simulations using a modified field map in which longitudinal fields are set to zero (${E}_{z}=0$) are compared with those using the original field map (${E}_{z}\ne 0$). The simulated longitudinal distribution at the position half-way between the two deflectors is shown while streaking. The calculated longitudinal resolution for these simulations is in the range 0.16 fs to 0.18 fs.

The effect of the induced energy spread within the TDS on the reconstruction has been studied by simulating a reconstruction in ASTRA with the longitudinal electric fields in the field map artificially set to zero at all positions within the two TDSs. Although this structure is unphysical, it serves as a useful model for seeing how the longitudinal fields influence the measurement. Figure 11 shows reconstruction results, using both a modified field map with the longitudinal fields set to zero and with the original map. In addition, results are shown for simulations including and excluding space-charge forces from the first TDS entrance, with the quadrupole strengths optimized for both cases. Both TDSs were used with a 20 MV peak voltage, operated at zero crossing, as for the simulations in Fig. 8. The rms bunch lengths of these profiles are displayed above. ${\sigma }_{t}^{{\rm{meas}}}$ is the result of the simulated measurement, including the subtraction of the spot size at the screen with the TDS off.

It can be seen that the longitudinal profile is influenced by the longitudinal electric fields in the structures, indicating that these fields are a key source of error in the measurement. The relative energy spread at the end of the second TDS where the longitudinal fields are set to zero is 0.005, compared to 0.013 when using the original field map. As well as affecting the longitudinal beam profile, these forces lead to coupling between the transverse and longitudinal planes, making the reconstruction of the profile less accurate. The reconstruction without space-charge forces and without longitudinal electric fields in the TDSs agrees well with the profile and appears to be limited mainly by the longitudinal resolution resulting from the original transverse size. The reconstruction including space-charge forces but with longitudinal forces in the TDSs set to zero also reproduces the shape of the distribution well but the width is much greater than the result from simulations without space-charge forces. A larger width is to be expected as the betatron beam size is anyway larger due to space-charge forces; however, it can be seen that even the rms bunch length calculated with subtraction of the spot size with the TDS off significantly overestimates the actual bunch length. One reason for this discrepancy is that the defocusing effect resulting from space-charge forces does not affect the shear parameter calculated from the phase scan as here only the mean position of the bunch is recorded, whereas it does affect the mapping to the beam profile at the screen. In addition, longitudinal space-charge forces may cause bunch-length elongation within the TDSs, influencing the measurement.

## 3D Charge-Density Reconstruction

The novel design of the PolariX-TDS allows bunches to be streaked at any transverse angle, in contrast to existing deflection structures. An image of a bunch on a screen after streaking is in effect a projection of the charge distribution onto the 2D plane spanned by the transverse axis perpendicular to the streaking direction and the longitudinal axis at the TDS. In this measurement, bunches are streaked at several transverse angles and the resulting projections are combined to form a full 3D charge-density distribution. The projections are each divided into longitudinal slices depending on the resolution and a 2D charge-density image is reconstructed for each slice using tomographic reconstruction techniques. Here, the Simultaneous Algebraic Reconstruction Technique (SART)^30^, using the Kaczmarz iterative solver method^31^, has been applied with two iterations. The procedure for this measurement has been proposed and detailed in earlier work^4^.

In this section, the feasibility of applying this measurement to ultrashort bunches at the ARES linac is considered. For the first time, ASTRA simulations are presented in order to test the process with the simulated field map of the structures and to study the impact of space-charge forces for the ARES parameter range. The simulated bunch distribution at the screen with the TDS off is compared to the reconstructed distribution, where the longitudinal information of the reconstruction pertains to the TDS position and the transverse information pertains to the screen position.

In order to achieve an accurate reconstruction, the bunch length of interest must be greater than the longitudinal resolution, as discussed above. As the longitudinal resolution depends on the transverse beam properties, it will vary depending on the streaking angle for transversely asymmetrical bunches and will be limited by the worst case. There are two quadrupoles upstream of the TDSs and two downstream that could be used for matching. In the simulations of the 1D reconstruction presented in the previous section, the two quadrupoles between the deflectors and the screen were used for matching. This approach is not possible, however, for a 3D reconstruction as it is important that the transfer function from the TDS to the screen in the axes parallel and perpendicular to streaking are decoupled. This is not the case when using two fixed quadrupoles in the lattice: unless streaking along the vertical or horizontal axes, there will be coupling between the transverse planes^32^.

The alternative of using the two quadrupoles before the TDS and leaving a drift between the TDS and the screen, however, has the main disadvantage that matching the quadrupoles to obtain a sufficiently fine longitudinal resolution is challenging due to space-charge effects. Of course, modifying the bunch distribution using quadrupoles before the TDSs to suit matching requirements also perturbs the charge density of the bunches. For these reasons, 3D charge-density reconstructions are a lot more challenging than the 1D measurement presented in the previous section.

**Figure 12:**
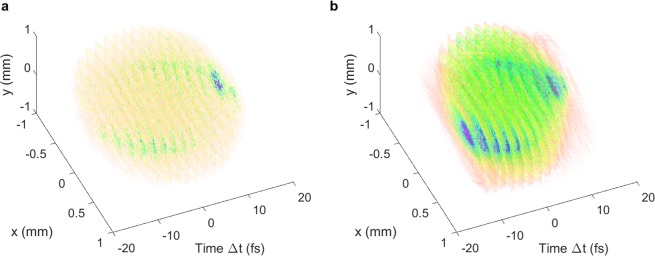
3D charge-density reconstruction in ASTRA with space-charge forces included in simulations for the few-femtosecond rms bunch. The distribution tracked to the screen with the TDS off (**a**) is shown alongside the distribution of the simulated reconstruction (**b**), where the transverse information pertains to the screen position and the longitudinal information to the TDS centre. The longitudinal distribution is sliced according to the longitudinal resolution of 2 fs for the reconstruction.

In the simplest case, the bunch is long enough compared to the resolution that no quadrupoles must be employed. This is the case for the first example in this section: a 5 fs rms bunch at the TDS position. The parameters for this bunch are shown in Table 4. In this case, the longitudinal resolution is approximately 2 fs including space-charge forces, calculated using Eq. 9 in the $x$-plane, as this is the plane with the largest rms spot size at the screen with the TDS off. Figure 12 shows a simulated 3D reconstruction measurement for this bunch using 16 streaking angles. For these simulated measurements, the fields inside each TDS are scaled to effect a kick of roughly 20 MV peak voltage on the bunch, with the phase set to zero crossing, as in the previous section. It is assumed that the accelerator provides perfect shot-to-shot stability, i.e. identical bunches have been used for all directions of streaking. The rms bunch length is 5.0 fs between the TDSs and 7.6 fs at the screen when the TDS is turned off so there is a discrepancy in the longitudinal profile when comparing the reconstruction and the simulated distribution at the screen. It can be seen that the general shape of the bunch is reconstructed, in particular the significant $x$-$t$ correlation.

**Table 4:** Bunch properties at the exit of the chicane and in between the two structures with the TDS and quadrupoles off for the few-femtosecond bunch. Quoted emittances are normalized.

Parameter	Chicane exit	Between structures
Mean energy (MeV)	84.2	84.2
Bunch charge (pC)	3.0	3.0
Bunch length rms (fs)	5.15	5.05
Rel. rms energy spread	0.0022	0.0018
Hor. rms beam size (mm)	0.088	0.157
Ver. rms beam size (mm)	0.097	0.137
Hor. emittance (mm mrad)	0.224	0.370
Ver. emittance (mm mrad)	0.190	0.271
Horizontal $\beta$-function (m)	5.7	11.0
Vertical $\beta$-function (m)	8.1	11.4

An exact reconstruction is not possible for several key reasons in addition to those mentioned in the 1D reconstruction discussion. First, the longitudinal profile is reconstructed at the centre of the TDS while the transverse information pertains to the screen position. At ultrarelativistic velocities this is not an issue; however, at energies within the typical ARES range the change in longitudinal profile may be significant, as seen in the previous section. Second, space-charge forces are dependent on the charge-density distribution, which is modified when the bunch is streaked. Lastly, there is always an inherent error involved in a tomographic reconstruction with a finite number of projections. In experiment, there will also be some variation in the distribution between shots; the effects of jitter on the final bunch parameters at ARES have been studied previously^27^. If necessary, shots could be selected based on feedback from the machine settings.

**Figure 13:**
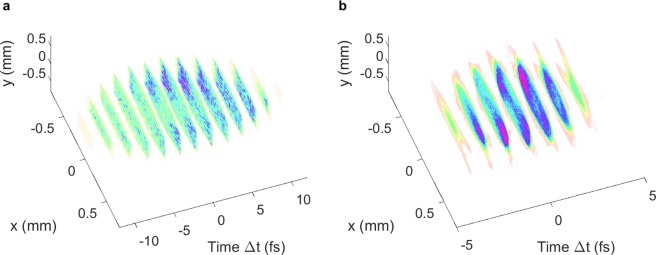
3D charge-density reconstruction in ASTRA with space-charge forces included in simulations for the subfemtosecond rms bunch. The distribution tracked to the screen with the TDS off (**a**) is shown alongside the distribution of the simulated reconstruction (**b**), where the transverse information pertains to the screen postion and the longitudinal information to the TDS centre. The longitudinal distribution is sliced according to the longitudinal resolution of 1.1 fs for the reconstruction.

The subfemtosecond bunch of WP1 is very challenging to reconstruct and the two quadrupoles upstream of the TDSs must be used to achieve a sufficient longitudinal resolution. An ASTRA scan over the two quadrupole strengths upstream of the TDSs was performed and the ratio of the spot size at the screen with the TDS off and the spot size with the TDS on in both the $x$ and $y$ planes was found including space-charge effects in the simulations. The quadrupole strengths that produced the minimum ratio for the maximum of the two planes were found to be $-6.6\ {{\rm{m}}}^{-2}$ and 5.9 ${{\rm{m}}}^{-2}$, for which a longitudinal resolution of 1.1 fs was obtained. For comparison, a longitudinal resolution of 0.4 fs would be possible if space-charge forces could be neglected. The simulated 3D reconstruction measurement for this bunch, including space-charge forces, is shown in Fig. 13. The rms bunch length is 1.0 fs between the structures and 5.1 fs at the screen when the TDS is turned off so there is again a discrepancy in the longitudinal direction between the reconstruction and the tracked distribution at the screen. For this bunch the reconstruction is especially limited by the longitudinal resolution and much less information may be deduced about this bunch than about the few-femtosecond rms bunch, although some qualitative features of the bunch are apparent in the reconstruction.

## Calibration, Manufacturing Imperfections and Jitter

The shear parameter is determined from the gradient of a plot of centroid displacement on the screen against phase. The phase scan will be affected by jitter in both the RF phase and the arrival time of the bunches. The minimum expected rms arrival time jitter with respect to a reference for bunches compressed in the magnetic chicane is less than 10 fs^33^. At the X-band frequency of operation, this would be equivalent to an rms phase jitter of 0.04°. The measured rms RF phase jitter for the X-band deflector at the Linac Coherent Light Source (LCLS) is 0. 1°^9^, which gives a guide to the value that can be expected eventually at ARES. The RF phase jitter and the arrival time jitter combined will lead to a noisy phase scan, increasing the uncertainty on the shear parameter and thereby introducing a systematic error into the measurement. In principle, the RF phase at the expected arrival time for each shot could be recorded with feedback from the RF system, significantly reducing the noise in the scan. Furthermore, the uncertainty on the shear parameter may be mitigated by increasing the phase range scanned, but there are limitations on this range that must also be considered—namely, the aperture constraints of the structures, the dimensions of the screens and the linearity of the phase-displacement relationship.

The aperture of the TDSs is limited by the irises to $\pm 4$ mm. Using the field maps introduced above, the phase range achievable using two TDSs at 20 MV per TDS is around ±0.5°. There are two screens on the straight diagnostic line following the TDSs—one directly after the second structure and the other at the end of the beamline after the two quadrupoles—which will have transverse dimensions of 30.5 mm by 24 mm. With a drift between the TDS and the screen, for a kick strength of 20 MV per structure, the second screen should intercept 100 MeV bunches with a phase range of approximately ±0.2°. Using quadrupoles matched as above, this range is considerably larger. Within the range given by the TDS aperture, the phase-displacement relationship is not greatly divergent from linear.

A further source of error that has not been considered so far is manufacturing imperfections in the dimensions of the cups that make up the structures. These will affect the phase advance in the TDSs and therefore lead to deviations in the expected trajectory of the beam. It is possible to compensate such effects by changing the set point of the temperature and the phase, although precise control of these parameters is therefore necessary. A study was conducted to check the impact of these manufacturing imperfections and the compensation possible, using a similar procedure to that applied to the TDS at the FERMI light source^34^. A random error distribution was generated with a maximum integrated phase advance error of 5° over the whole structure and a standard deviation of 1°, which corresponds to a precision of approximately $3.5\ {\rm{\mu }}{\rm{m}}$ in the manufacturing of the cells, although it is hoped that the values achieved will be considerably better than this. The effects of the matching cells on the trajectory discussed above were not taken into account in this study. The phase advance shift due to temperature is given by $\Delta \phi =c/{v}_{{\rm{g}}}\ {\alpha }_{T}\ {\phi }_{0}\ {\rm{d}}T$, where ${v}_{g}$ is the group velocity. For this specific error distribution, the second TDS must be operated at a temperature 0.3 °C cooler than the first and at a phase of −3.17° with respect to the first, which compensates these errors almost entirely.

**Figure 14:**
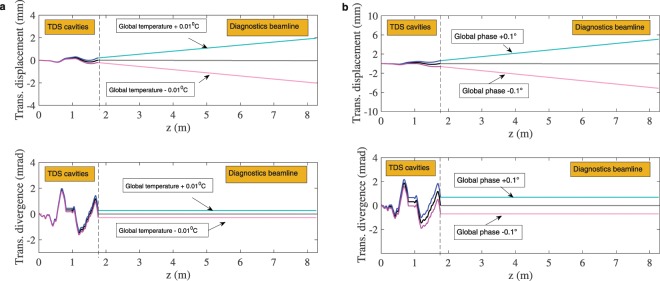
Sensitivity to temperature (**a**) and phase (**b**) deviations from the corrected values for a random distribution of errors with a maximum integrated phase advance error of 5° over the whole structure and a standard deviation of 1°. The deviations considered are the same for the two TDSs. Quadrupoles are assumed to be turned off.

As the temperature a/nd phase are used for correction of both the manufacturing imperfections and the kicks in the matching cells, it is important that they can be set precisely and that the jitter is low. Figure 14 shows the effect of a deviation from the corrected temperature and phase on the transverse position and divergence without considering quadrupoles in the beamline. The temperature and phase are varied independently and the same deviation is assumed for both deflectors. From these studies, the temperature and phase corresponding to a maximum offset of $\pm$1 mm within the structures with the given input random error distribution are calculated to be $0.03{5}^{\circ }{\rm{C}}$ and $0.1{5}^{\circ }$ respectively. The temperature and phase corresponding to a deviation of $\pm$5 mm at the second screen without quadrupoles are 0.025 °C and 0.17° respectively.

Based on the considerations above, a temperature jitter and stability of less than $0.0{2}^{\circ }{\rm{C}}$ and a phase jitter and stability of around $0.{1}^{\circ }$ should be aimed at. Even if these are achieved, the beam will still jitter considerably.

A commonly-used technique to minimize the error on the shear parameter is to perform a phase scan at a lower deflection voltage, at which a larger phase range can be accommodated. The shear parameter can then be scaled up linearly according to Eq. (7). In experiment, the voltage will not be measurable directly but the input power will be known. The voltage scales as the square root of power, assuming a constant shunt impedance. The first screen may be used to calibrate the scaling between voltage and input power.

## Conclusions and Outlook

Transverse deflection structures are already widely used in linear electron accelerators for diagnostic purposes and provide very useful information about beam properties during the operation of machines. Future accelerators, such as the ARES linac, are aiming to produce ultrashort bunches of femtosecond and subfemtosecond duration for external injection into novel accelerating structures. Such bunches are shorter than any other bunches that have been measured previously with a TDS, and their measurement presents particular challenges. These challenges have been explored and discussed with the help of simulations, which include both the simulated field map of the TDS and 3D space-charge forces. The studies show, for the first time, that it should be possible to reconstruct the longitudinal profiles of subfemtosecond bunches and measure their rms bunch lengths with the setup envisaged for ARES with a resolution down to 0.2 fs. This value for the resolution incorporates the effects of the finite transverse beam size; however, there are a number of different effects that also contribute to discrepancy in the reconstruction, which have been investigated in this paper. These various sources of uncertainty, including the effect of the longitudinal fields within the TDS, have been studied with detailed simulations, and it has been shown that, despite these undesired effects, an impressive reconstruction of the longitudinal profile should be possible at ARES. In addition, simulations have been presented that show how the two TDSs could be used independently to measure the change in bunch length between them, from which important information about the behaviour of the bunch downstream of the TDS may be deduced.

As well as these measurements, the PolariX-TDS allows bunches to be streaked at any transverse angle, which makes it possible to reconstruct their 3D charge-density distribution. The same complications as in the longitudinal-profile reconstruction also manifest themselves in this measurement, which is further complicated by the fact that the transverse and longitudinal information in the reconstruction pertain to different positions in the beamline. In this paper, realistic ASTRA simulations of virtual measurements have been presented for the first time in this range of beam parameters, and it has been shown that, despite the difficulties, it is possible to reconstruct the 3D charge-density distribution of a bunch a few femtoseconds long and obtain useful qualitative information, for example about the shape of the distribution and correlations between planes. The flexibility in the lattice allows further interesting measurements. The two quadrupoles before the TDS could be used, for instance, to simulate the matching into the plasma, which will eventually be carried out with three permanent magnetic quadrupoles.

This paper has focused on the reconstruction of the charge-density distribution, both in 1D and 3D; however, other phase-space information may also be retrieved, such as the longitudinal phase space^35^ or the slice emittance. Further simulation studies will investigate these measurements in more detail and consider how to combine the data from these measurements in order to characterize bunches as completely as possible.

The prototype PolariX-TDS has been successfully commissioned in the FLASHForward beamline, and it has been shown to operate very well^36,37^. The ARES linac, however, will operate at a lower energy and with much shorter bunches, creating additional challenges, which will be fully investigated experimentally when the TDSs are installed in the beamline there in 2020.

## Data Availability

The simulation data are available from the corresponding author on reasonable request.
